# *Sphingobacterium *respiratory tract infection in patients with cystic fibrosis

**DOI:** 10.1186/1756-0500-2-262

**Published:** 2009-12-23

**Authors:** Antonietta Lambiase, Fabio Rossano, Mariassunta Del Pezzo, Valeria Raia, Angela Sepe, Fabiola de Gregorio, Maria Rosaria Catania

**Affiliations:** 1 Department of Cellular and Molecular Biology and Pathology "Luigi Califano", Medicine School, University of Naples "Federico II", Pansini street, 80131, Naples, Italy; 2 Department of Pediatrics, Regional Cystic Fibrosis Center, Medicine School, University of Naples "Federico II", Pansini street, 80131, Naples, Italy

## Abstract

**Background:**

Bacteria that belong to the genus *Sphingobacterium *are Gram-negative, non-fermentative bacilli, ubiquitous in nature and rarely involved in human infections. The aims of this study were to evaluate the epidemiology of infection by *Sphingobacterium *in a cohort of patients affected by Cystic Fibrosis (CF), the antibiotic susceptibility and the DNA fingerprinting of the isolated strains and to analyze some clinical outcomes of the infected patients.

**Findings:**

Between January 2006 and June 2008, patients (n = 332) attending the Regional CF Unit in Naples, Italy, were enrolled.

Sputum samples were processed for microscopic, cultural, phenotypic identification and antibiotic susceptibility testing. DNA fingerprinting was performed by pulsed-field gel electrophoresis (PFGE). A total of 21 strains of *Sphingobacterium *were isolated from 7 patients (13 of *S. spiritovorum*, 8 of *S. multivorum*). *S. multivorum *isolates were more resistant than those of *S. spiritovorum*. PFGE profiles were in general heterogeneous, which suggested independent circulation.

**Conclusions:**

This is the first Italian report about respiratory tract infections by *Sphingobacterium *in CF patients. In our cohort, these infections were not associated with a deterioration of pulmonary function during the follow-up period. Although the exact role of this microorganism in CF lung disease is unknown and the number of infected patients was small, this study could represent an important starting-point for understanding the epidemiology and the possible pathogenic role of *Sphingobacterium *in CF patients.

## Findings

In Cystic Fibrosis (CF) patients, bronchiectasis and obstructive pulmonary disease are the primary causes of morbidity and mortality [1]. As pulmonary disease therapy and nutritional care have improved life expectancy, new emerging pulmonary pathogens, such as bacteria of the *Burkholderia cepacia *complex, *Alcaligenes xylosoxidans*, *Stenotrophomonas maltophilia *and nontuberculous mycobacteria have been detected in CF patients [2-8].

Bacteria that belong to the genus *Sphingobacterium *are Gram-negative, non-fermentative microorganisms that are positive for catalase and oxidase tests. They are ubiquitous in nature, but are rarely involved in human infections. An important feature of bacteria that belong to this genus is the presence of high concentrations of sphingophospholipids in the cellular lipid components, from which the name *Sphingobacterium *is derived. The genus *Sphingobacterium *consists of six species: *S. spiritovorum*, *S. multivorum*, *S. mizutaii*, *S. antarcticum*, *S. faecium *and *S. thalpophilum*. The species *S. multivorum *and *S. spiritovorum *have been associated with bacteremia, peritonitis, and chronic respiratory infection in patients with severe underlying conditions [9-13].

In the last years, some strains of *Sphingobacterium *have been isolated from respiratory samples of CF patients attending our Regional CF Center.

The finding of these microorganisms in our CF patients led us to investigate this phenomenon. Therefore, in this study, we investigated the prevalence of infection by *Sphingobacterium *in a cohort of CF patients and examined the antibiotic susceptibility of these strains. In addition, we performed DNA fingerprinting and analyzed some clinical outcomes of patients infected by these bacteria.

## Methods

### Study population

Microbiological samples (sputum) were obtained during the period January 2006 to June 2008 from 332 CF patients (147 males, 185 females; mean age 18.15 years; range 0.5-52 years) who were regularly attending the Regional Referral CF Center of Naples. CF diagnosis was confirmed by standard methods (sweat chloride concentration above 60 mmol/L) and by genetic analysis.

At each visit, body weight and body mass index, number of episodes of pulmonary exacerbation, number and duration of hospital admissions required for pulmonary exacerbation and number of intravenous administrations of antibiotics were registered on an electronic case report form. Patients over 6 years of age had at least one evaluation of lung function per year as measured by forced expiratory volume in 1 sec (FEV_1_), and expressed as a percentage of predicted values for the relevant age, sex, ethnic background, weight and height.

Sputum samples for microbiological studies were obtained from each patient at least quarterly at clinical examinations.

Chronic lung infection was defined as the persistence of pathogen in three positive cultures for at least six consecutive months [14]. Co-infection was defined as the presence of more than one microorganism in the sputum sample.

Patients infected by *Sphingobacterium *were characterized for age, age of acquisition of first infection, co-infection and lung function.

Multidrug resistance was assessed according to the guidelines of the CF Foundation for *P. aeruginosa *[15], which were extended to all *Sphingobacterium *isolates.

This study was conducted in agreement with the Declaration of Helsinki.

### Processing of samples, culture and identification of microorganisms

Sputum samples obtained from all patients during the study period were mixed with an equal volume of 1% dithiothreitol before incubation at 37°C for 30 min. All specimens were examined microscopically and plated on several agar media, which included blood agar, Muller-Hinton agar, MacConkey agar, *Burkholderia cepacia *selective agar (BCSA) and Sabouraud agar, at 37°C for up to 72 h.

All isolates obtained from the samples were identified using the Phoenix system (Becton Dickinson) and, for the identification of Gram-negative non-fermentative bacteria, the API 20 NE identification system (bioMérieux) was also used.

Isolates that were identified as *S. multivorum *or *S. spiritovorum *were processed for DNase, oxidase, urease activity and motility tests [16].

The DNase activity was tested using the method of O'Brien and Davis [17]: briefly, DNase test agar (DNase agar CM321, Oxoid) that contained 0.01% (vol/vol) toluidine blue was used. The plates were examined for pink zones around the colonies. In the absence of dye, the plates were flooded with 1 N HCl, allowed to stand on the bench (lids uppermost) for a few minutes and then examined for clear zones around the colonies. For the oxidase test, Taxo Discs (BBL™) were used. For the urease test, Urea Agar Slants (BBL™) were used. For the motility test, colonies were inoculated into Motility GI Medium tubes (Difco) and incubated at 37°C for 18 h. Motility was indicated by diffuse bacterial growth away from the inoculum zone.

### Antibiotic-susceptibility test

To assess the sensitivity of the *Sphingobacterium *isolates to antibiotics, an agar-diffusion method (Kirby-Bauer) and a microbroth dilution assay (Phoenix) were used. For all the methods used in the study, interpretative criteria for susceptibility to antibiotics were in accordance with National Committee for Clinical Laboratory Standards criteria [18]. The following drugs were assayed: amikacin, aztreonam, piperacillin, piperacillin-tazobactam, cefotaxime, cefepime, ceftazidime, ciprofloxacin, levofloxacin, chloramphenicol, imipenem, meropenem, trimethoprim-sulfamethoxazole and gentamicin.

### Genotyping by PFGE

DNA fingerprinting was carried out by the method described by Grothues *et al*. [19]. Briefly, isolates were grown overnight and suspended in SE buffer (75 mM NaCl, 25 mM EDTA, pH 7.5). The cell suspensions (4 McFarland units) were mixed with an equal volume of 1.6% low-melting point agarose, molded into plugs at 4°C, and lysed with lysis buffer (1% N-lauryl sarcosine, 0.5 M EDTA, pH 8) to which Proteinase K (500 μg/mL) had been added. The DNA inserts were digested by *ApaI *(New England Biolabs), in accordance with the manufacturer's instructions. Macrorestriction fragments were separated using the CHEF-DR III PFGE system (Bio-Rad) at 10°C for 20 h, with an initial switch time of 5 s and a final switch time of 35 s, at a field strength of 6 V/cm. A ladder of lambda phage DNA concatemers was used as a size marker. Fragment patterns were compared according to the criteria described by Tenover [20]. Based on these criteria, we considered isolates to be possibly related if their restriction patterns differed by 4-6 bands and closely related if their restriction patterns differed by no more than 2-3 bands. Isolates were considered different if their restriction patterns differed by 7 or more bands.

## Results

### Infected and chronically-infected patients, co-infection with other microorganisms

During the study period, a total of 21 isolates of *Sphingobacterium *was detected (8 isolates of *S. multivorum *and 13 isolates of *S. spiritovorum*) from 7 of the 332 patients enrolled (2.1%; 2 males, 5 females). *S. spiritovorum *was isolated from four patients and *S. multivorum *was isolated from three patients. Among these, one patient was identified as chronically infected by *S. multivorum*.

All patients were co-infected by at least one other Gram-negative pathogen, and in four patients, co-infection by Gram-positive bacteria was also found (as indicated in table 1).

**Table 1:** Co-infection with other bacteria, type of CFTR mutation and pancreatic insufficiency in patients with *Sphingobacterium *infection

Strains	Number of patients	Co-infection with other bacteria	CFTR mutation	Number of patients with PI
*S. spiritovorum*	4 (3 Females/1 Male)	P. aeruginosa (1 patient), P. aeruginosa+S. maltophilia+S. aureus (1 patient), P. aeruginosa+S. aureus (2 patients)	ΔF508 (2 patients), ΔF508/Ot (1 patient), Ot/Ot (1 patient)	4

*S. multivorum*	3 (2 Females/1 Male)	P. aeruginosa+S. marcescens (1 patient), S. marcescens (1 patient), P. aeruginosa+S. aureus (1 patient)	ΔF508 (1 patient), ΔF508/Ot (1 patient), Ot/Ot (1 patient)	3

Ot: Other;

PI: pancreatic insufficiency

### Identification of *S. multivorum *and *S. spiritovorum*

These bacteria showed growth on blood agar, on which they appeared as convex, smooth, opaque, non-hemolytic colonies of 1 mm in diameter after 48 h of incubation at 37°C. Although they appeared as small yellow colonies on Mueller-Hinton medium after 24 h of incubation at 37°C, no growth or limited growth was observed on MacConkey agar. The bacteria did show growth on BCSA after 48 h of incubation at 37°C.

All 21 isolates of *Sphingobacterium *were non-motile and positive for DNAse, oxidase and urease tests.

The automated system used for identification had a reliability level of 99% for the above mentioned bacteria and identification was confirmed by the API 20 NE system.

### Antibiotic-susceptibility test

On the basis of the criteria applied in this study, all the *S. multivorum *isolates showed multidrug resistance, whereas the *S. spiritovorum *isolates were not considered multiresistant. In particular, the *S. multivorum *isolates were resistant to β-lactams, including carbapenems and aminoglycosides; only trimethoprim-sulfamethoxazole (minimum inhibitory concentration-MIC<0.5/9.5 μg/mL) and quinolones (ciprofloxacin, MIC<0.5 μg/mL; levofloxacin, MIC<1 μg/mL) were active against these strains. The *S. spiritovorum *isolates showed sensitivity to ceftazidime, carbapenems, trimethoprim-sulfamethoxazole and quinolones (as indicated in table 2).

**Table 2:** Percentage of *Sphingobacterium *strains resistant to the antibiotics tested.

	AMK	GEN	ATM	SXT	FEP	CTX	CAZ	MEM	IPM	CIP	LVX	PIP	TZP	CHL
*S. spiritovorum *(13)	100	100	100	0	100	100	0	0	0	0	0	0	0	100

*S. multivorum *(8)	100	100	100	0	100	100	100	100	62.5	0	0	100	100	100

AMK = Amikacin; GEN = Gentamicin; ATM = Aztreonam; SXT = Trimethoprim-sulfamethoxazole; FEP = Cefepim; CTX = Cefotaxim; CAZ = Ceftazidime; MEM = Meropenem; IPM = Imipenem; CIP = Ciprofloxacin; LVX = Levofloxacin; PIP = Piperacillin; TZP = Piperacillin/tazobactam; CHL = Chloramphenicol.

### Genome macrorestriction analysis

**Figure 1:**
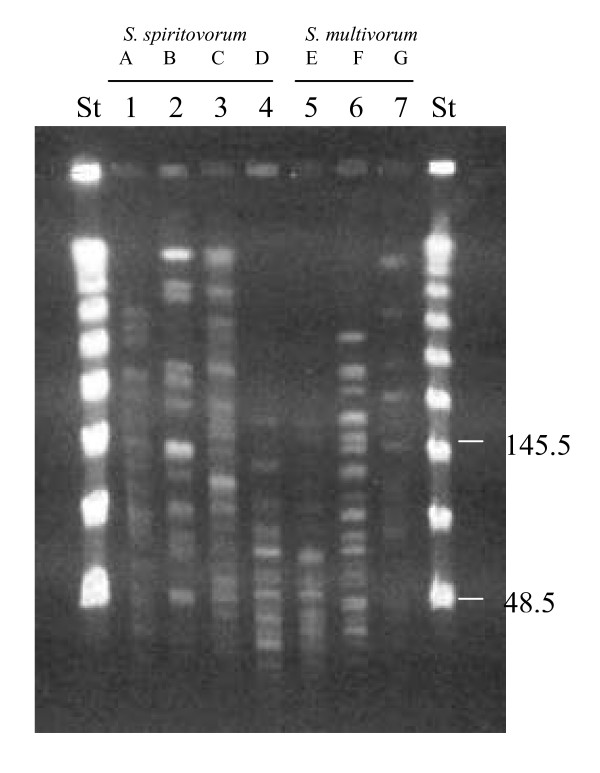
**PFGE profiles of genomic DNA digested with *ApaI *of *Sphingobacterium *strains**. From lanes 1 to 4: *S. spiritovorum *strains from A to D profiles, respectively. From lanes 5 to 7: *S. multivorum *strains from E to G profiles, respectively. Molecular size markers (a ladder of lambda phage DNA concatemers) were run in lanes St. Sizes are indicated in kilobases.

PFGE analysis showed a high heterogeneity of restriction patterns. All 7 patients were infected by strains that were epidemiologically different (strains A-G) (Fig. 1). Moreover, sequential isolates taken from same patient had identical PGFE profiles.

### Clinical manifestations

Of the 7 patients infected by *Sphingobacterium *(mean age 10.8 years, range 4-24 years) all showed pancreatic insufficiency at CF diagnosis. The mean age at diagnosis was 13.4 months (range 5 months-22 years), whereas the mean age at first acquisition of *Sphingobacterium *infection was 6.5 years (range 1-15.5 years). The results of the genetic analysis showed that three of the patients were homozygous for the ΔF508 mutation, two were heterozygous for ΔF508 and another mutation and two were heterozygous for other mutations (see table 1).

During the follow-up period (mean period of 14.4 months, range 7 months to 3 years), no variation in FEV_1 _values (mean FEV_1 _72.3%; range 43-91) or increase in acute lung infections were found in our cohort of patients.

## Discussion

The species of *Sphingobacterium *that were identified in this study were *S. multivorum *(formerly *Flavobacterium multivorum*, CDC group Ilk-2), and *S. spiritovorum *(includes species formerly designated *Flavobacterium spiritovorum*, *Flavobacterium yabuuchiae*, CDC group Ilk-3) [16].

Apart a previous anecdotic report in 1992 [12], this is the first Italian report of the clinical and microbiological analysis of *Sphingobacterium *strains isolated from a cohort of patients who are regularly attending a Regional CF Unit.

The existence of bacterial biodiversity in the CF lung is well known [21] and, consequently, the correct identification of different bacterial species is important. Given that commercial systems for the phenotypic identification of non-fermentative Gram-negative bacteria recovered from CF patients appear to have some limitations, the isolation of bacteria with selective media is crucial for the processing of sputum samples from CF patients. In addition, the CF community, the Centers for Disease Control and Prevention and the American Society for Microbiology endorse the use of selective media for processing respiratory tract specimens from CF patients [22]. The selective medium used in this study was BCSA and the *Sphingobacterium *strains were able to grow on this medium. It is interesting to note that BCSA contains gentamicin (0.01 g/L), and the strains described were gentamicin resistant. Nevertheless, Yabuuchi reported that some strains of *Sphingobacterium *are susceptible to gentamicin [16].

In this study, we confirmed the phenotypic identification of *Sphingobacterium *strains, using the Phoenix and API 20 NE systems, by other tests (DNase, oxidase, urease activity and motility tests), as described by Yabuuchi [16].

No data are available about antibiotic resistance and/or susceptibility in Sphingobacteria in CF patients. In our study the results of the antibiotic-susceptibility profile of the strains showed that trimethoprim-sulfamethoxazole, ciprofloxacin, and levofloxacin were effective against the *Sphingobacterium *strains, in agreement with previous reports [9,23]. Considering that all patients are co-infected by other bacteria, we suggest that antibiotic treatment should be performed in pulmonary exacerbation. We found that the strains showed a high degree of clonal heterogeneity as demonstrated by PFGE, which yielded 7 different pulsed-types. It is noteworthy that these bacteria are found in the natural environment and, therefore, studies on microbiological cultures and typing of *Sphingobacterium *from hospital environments are ongoing. On the basis of our PFGE data, there is no evidence that *Sphingobacterium *can spread from one patient to another in our CF Center. However, a comprehensive epidemiological survey is mandatory.

No subsequent decline in lung function was registered. However, the follow-up period was too short to evaluate the clinical impact of *Sphingobacterium *in CF patients. To date, no further information is available about the role of these bacteria during the progression of pulmonary disease in CF patients; neither, there is sufficient evidence to define this microorganism as an emerging pathogen in CF patients.

This pilot study suggests that correct identification, followed by antibiotic-susceptibility testing and DNA-fingerprinting of all bacteria isolated from respiratory samples of CF patients are necessary in order to monitor the potential impact of chronic lung infection on clinical outcome.

## Competing interests

The authors declare that they have no competing interests.

## Authors' contributions

AL coordinated data analysis and drafted the paper. FR participated in the study design and coordination. MDP coordinated the analysis of clinical specimens. VR coordinated data relating to patients. AS collected data relating to patients. FDG collected data relating to patients. MRC conceived the study design. All authors read and approved the final manuscript
